# Identification, characterization and heparin binding capacity of a spore-wall, virulence protein from the shrimp microsporidian, *Enterocytozoon hepatopenaei* (EHP)

**DOI:** 10.1186/s13071-018-2758-z

**Published:** 2018-03-12

**Authors:** Pattana Jaroenlak, Dominic Wiredu Boakye, Rapeepun Vanichviriyakit, Bryony A. P. Williams, Kallaya Sritunyalucksana, Ornchuma Itsathitphaisarn

**Affiliations:** 1 Department of Biochemistry, Faculty of Science, Mahidol University, Bangkok, Thailand; 2 Center of Excellence for Shrimp Molecular Biology and Biotechnology (Centex Shrimp), Faculty of Science, Mahidol University, Bangkok, Thailand; 3 Biosciences, College of Life and Environmental Sciences, University of Exeter, Devon, UK; 4 Department of Anatomy, Faculty of Science, Mahidol University, Bangkok, Thailand; 5 National Center for Genetic Engineering and Biotechnology (BIOTEC), National Science and Technology Development Agency (NSTDA), Pathumthani, Thailand; 6 Shrimp Pathogen Interaction Laboratory (SPI), National Center for Genetic Engineering and Biotechnology (BIOTEC), Bangkok, Thailand

**Keywords:** EHP, *Enterocytozoon hepatopenaei*, Spore wall protein, SWP, Heparin, Heparin binding protein

## Abstract

**Background:**

The microsporidian *Enterocytozoon hepatopenaei* (EHP) is a spore-forming, intracellular parasite that causes an economically debilitating disease (hepatopancreatic microsporidiosis or HPM) in cultured shrimp. HPM is characterized by growth retardation and wide size variation that can result in economic loss for shrimp farmers. Currently, the infection mechanism of EHP in shrimp is poorly understood, especially at the level of host-parasite interaction. In other microsporidia, spore wall proteins have been reported to be involved in host cell recognition. For the host, heparin, a glycosaminoglycan (GAG) molecule found on cell surfaces, has been shown to be recognized by many parasites such as *Plasmodium* spp. and *Leishmania* spp.

**Results:**

We identified and characterized the first spore wall protein of EHP (EhSWP1). EhSWP1 contains three heparin binding motifs (HBMs) at its N-terminus and a Bin-amphiphysin-Rvs-2 (BAR2) domain at its C-terminus. A phylogenetic analysis revealed that EhSWP1 is similar to an uncharacterized spore wall protein from *Enterospora canceri.* In a cohabitation bioassay using EHP-infected shrimp with naïve shrimp, the expression of *EhSWP1* was detected by RT-PCR in the naïve test shrimp at 20 days after the start of cohabitation. Immunofluorescence analysis confirmed that EhSWP1 was localized in the walls of purified, mature spores. Subcellular localization by an immunoelectron assay revealed that EhSWP1 was distributed in both the endospore and exospore layers. An in vitro binding assay, a competition assay and mutagenesis studies revealed that EhSWP1 is a *bona fide* heparin binding protein.

**Conclusions:**

Based on our results, we hypothesize that EhSWP1 is an important host-parasite interaction protein involved in tethering spores to host-cell-surface heparin during the process of infection.

**Electronic supplementary material:**

The online version of this article (10.1186/s13071-018-2758-z) contains supplementary material, which is available to authorized users.

## Background

Microsporidia are obligate, intracellular, spore-forming parasites and currently considered as a sister group to fungi [1]. Microsporidia are important pathogens that infect a wide range of animal hosts from beneficial invertebrate to vertebrate species [2, 3]. Since the discovery of the first microsporidian *Nosema bombycis* in silkworms in the nineteenth century [4], it remains the cause of a fatal disease referred to as Pébrine that causes economic losses in the sericulture industry [5, 6]. *Enterocytozoon hepatopenaei* (EHP) is a close evolutionarily relative of *Enterocytozoon bieneusi* and other human-infecting microsporidia in the genus *Encephalitozoon* that cause life-threatening diarrhea in immunocompromized humans [7]. In aquatic animals, infection of microsporidia in fish leads to reduction in growth rate and productivity [8], and this is true also for EHP in shrimp [9].

Microsporidia display many unique cellular and genetic characteristics. At the cellular level, microsporidia lack peroxisomes and a typical Golgi structure [10, 11]. Their mitochondria are structurally and functionally reduced into organelles called mitosomes [12, 13]. Their genomes are remarkably compact due to the loss of genes in metabolic pathways and reduction in intergenic spaces [14]. The 2.3 Mbp genome of *E. intestinalis* is the smallest eukaryotic genome known to date [15]. In addition, microsporidia have developed a characteristic invasion mechanism that involves the polar tube and the spore wall [16]. At the first step of infection, the spore wall proteins are capable of interacting with host cell glycosaminoglycans (GAGs) [17, 18]. Under suitable conditions, the polar tube is extruded to pierce the host cell membrane. This process rapidly occurs in less than 2 milliseconds [11, 19]. The polar tube then serves as a conduit to transfer an infectious sporoplasm into the host cell to begin the parasitic, intracellular phase of the life-cycle [11].

The spore walls of microsporidia consist of two layers, a proteinaceous electron dense exospore layer and a chitinous electron lucent endospore layer [20]. Many spore wall proteins (SWPs) are found in these layers [21]. They participate in the host cell recognition process and provide structural support for the spore wall [17, 21, 22]. SWPs have been extensively characterized for the genera *Nosema* and *Encephalitozoon*. These include NbSWP5, NbSWP11, NbSWP12, NbSWP16, NbSWP25 and NbSWP26 from *N. bombycis* [22–27], EcEnP1, EcEnP2 and chitin deacetylase (EcCDA) from *E. cuniculi* [28, 29], and EiEnP1 from *E. intestinalis* [18]. Recently, *Antonospora locustae* SWP2 (AlocSWP2) has been shown to be involved in sporulation [30].

Hepatopancreatic microsporidiosis (HPM) in cultivated shrimp is characterized by slow growth and wide size variation, making the causative agent *E. hepatopenaei* (EHP) an economically important pathogen for shrimp farmers [31, 32]. EHP was initially reported as a new, undescribed microsporidian in hepatopancreatic tissue of the black tiger shrimp *Penaeus monodon* in Thailand in 2004 [33], but it was not characterized and named as a new species until 2009 [34]. Thus, it was an endemic pathogen that was also able to cause disease in the exotic Pacific-white shrimp *P. vannamei* [35] that replaced *P. monodon* as the dominant and most economically important shrimp species cultivated in Thailand. Currently, EHP is known to occur widely in Asia (e.g. Thailand, China, India, Vietnam, Indonesia and Malaysia) and it has been reported more recently from Venezuela [34, 36–38]. In Thailand, EHP is now the third most serious problem for shrimp farmers after white spot disease (WSD) caused by white spot syndrome virus and acute hepatopancreatic necrosis disease (AHPND) caused by unique *Vibrio* isolates that produce Pir-like toxins [9].

Since EHP is a threat to the global shrimp industry, a better understanding of its infection mechanisms and virulence is urgently needed to facilitate the development of preventative and therapeutic strategies. Previously, a cohabitation assay revealed that EHP can be horizontally transmitted via water in shrimp cultivation ponds [39]. Thus, any treatment or management protocol that would stop or interfere with transmission would constitute an effective control measure. However, knowledge of how EHP interacts with the host is still poorly understood. This study therefore aimed at a better understanding of the process. From whole genome sequencing of EHP [40], the spore wall protein EhSWP1 was first identified and its gene sequence was used to develop a more specific PCR detection method called SWP-PCR [31]. Here, we functionally characterize EhSWP1, show that it contains three heparin binding motifs (HBMs) and one Bin-amphiphysin-Rvs-2 (BAR2) domain, that it is localized in the exospore and endospore layers, and that interacts with heparin via its HBMs. We hypothesize that EHP uses this recognition process to initiate host cell infection, and we hope that this understanding may lead to identification of vulnerable targets for development of preventative and therapeutic methods to control EHP in the shrimp aquaculture industry.

## Methods

### Shrimp and EHP specimens

With permission from the farm owners to collect specimens for this study from their properties, EHP-infected *P. vannamei* (7–10 g) were collected from commercial shrimp farms in Thailand. Hepatopancreata of EHP-infected shrimp were dissected as previously described [31] to obtain spores for purification by discontinuous Percoll gradient centrifugation [40]. The purified spores were washed with sterile distilled water and stored at room temperature.

### Bioinformatics analysis

In this study, we used predicted proteins encoded by the genomes of 23 microsporidian species (*Enterospora canceri*, *Enterocytozoon hepatopenaei*, *Hepatospora eriocheir*, *Hepatospora eriocheir canceri*, *Anncaliia algerae*, *Ordospora colligata*, *Trachipleistophora hominis*, *Spraguea lophii*, *Vittaforma corneae*, *Encephalitozoon romaleae*, *Vavraia culicis*, *Edhazardia aedis*, *Encephalitozoon hellem* Swiss, *Encephalitozoon hellem* ATCC, *Nematocida parisii* ERTm1, *Nematocida parisii* ERTm3, *Nematocida* sp*.* ERTm2, *Nematocida* sp. ERTm6, *Enterocytozoon bieneusi*, *Encephalitozoon intestinalis*, *Encephalitozoon cuniculi*, *Nosema bombycis* and *Nosema ceranae*). These were downloaded from public databases NCBI and MicrosporidiaDB. Ortholog clusters in which these proteins belonged were identified by initially querying the proteins from all 23 microsporidian genomes against their own database by using BLASTP with an e-value cut-off of 1e-03 [41]. An ortholog prediction program, ORTHOMCL on its default settings, was then used to convert the BLASTP output into ortholog clusters [42]. Phylogenetic assessment of the ortholog groups in which EHP SWPs were grouped was performed as follows. The proteins in the two ortholog groups in which EHP SWPs were clustered were first aligned with the online MAFFT program using the L-INS-I iterative refinement setting and then trimmed with GBLOCKS with less stringent settings (allowing smaller final blocks, gap positions in the final blocks and less strict flanking positions). A Bayesian inference method was also used to infer the phylogenetic relationship between the proteins in the ortholog clusters. Here, the trimmed alignment was passed to the online MR BAYES tool on the CIPRES online portal. MR BAYES was run using an LG+GAMMA model and default settings [43]. Subsequent phylogenetic analyses performed on the SWP12 clade were performed following the same protocols as explained above. Although EHP00_1468 did not cluster with any microsporidian protein in our ORTHOMCL analyses, we included it in our phylogenetic analyses as it had 98% identity to EHP00_350 in initial BLASTP analyses.

Conserved domains of proteins were predicted with MOTIF SCAN (http://www.genome.jp/tools/motif/). MOTIF SCAN searches protein sequences against a PFAM library of Hidden Markov Models (HMMs). To further assess the conservation of BAR2 domains within proteins in the SWP ortholog clusters, a pairwise alignment with the EMBOSS STRETCHER tool (https://www.ebi.ac.uk/Tools/psa/emboss_stretcher/) of each protein against the PFAM BAR2 consensus sequence was performed. This is the consensus alignment sequence of seed proteins used by PFAM for the construction of the BAR2 HMM. The complete PFAM seed library for various functional domains can be downloaded from ftp://ftp.ebi.ac.uk/pub/databases/Pfam/current_release/Pfam-A.seed.gz. Phosphorylation site prediction was carried out by SCANPROSITE tool (http://prosite.expasy.org/prosite.html). NETNGLYC (http://www.cbs.dtu.dk/services/NetNGlyc/) and NETOGLYC (http://www.cbs.dtu.dk/services/NetOGlyc/) were used to predict N- and O-glycosylation sites, respectively.

### Reverse transcription PCR (RT-PCR) analysis

To achieve EHP infections, naïve, uninfected, test *P. vannamei* were co-habitated with EHP-infected *P. vannamei* as previously described [39]. Briefly, naïve *P. vannamei* shrimp were kept in tanks containing 150 l artificial seawater (Mariscience Co. Ltd, Bangkok, Thailand) at 25 ppt and 28 °C with a basket cage containing EHP-infected *P. vannamei* in the center of the tank. At 0, 5, 7, 9, 11 and 20 days after cohabitation, shrimp were collected and their hepatopancreatic tissue was aseptically removed for RNA extraction. Total RNA was extracted using Ribozol RNA extraction reagent (Amresco, Philadelphia, USA) and used as template RNA in reverse transcription reactions employing ImPromp-II reverse transcriptase (Promega, Wisconsin, USA) to produce cDNA using an oligo-dT primer. cDNA was subsequently used as the template for standard PCR with Green PCR master mix containing Taq DNA polymerase (Biotechrabbit, Hennigsdorf, Germany). The full-length *EhSWP1* gene was amplified by specific primer pairs, EHP_SWP01_F; 5'-–ATA TCC ATG GGC ATG TTA GAA GAT GCA AAG-3' and EHP_SWP01_R; 5'-ATA TCT CGA GAG AAA ATT TTT CAA GGT G-3'. Specific primer pairs for the actin gene of *P. vannamei* (*PvActin*) were used as an internal control (Actin_F; 5'-CCT CGC TGG AGA AGT CCT AC3' and Actin_R; 5'-TGG TCC AGA CTC GTC GTA CTC-3') [31, 44]. The PCR protocol for both *EhSWP1* and *PvActin* was as follows: denaturation at 95 °C for 5 min followed by 30 cycles of 30 s denaturation at 95 °C, 30 s annealing at 55 °C and 45 s extension at 68 °C, with a final extension for 5 min at 68 °C. The expected PCR amplicons were 687 bp and 401 bp for *EhSWP1* and *PvActin*, respectively. The amplicons were analyzed by 1.5% agarose gel electrophoresis with ethidium bromide staining.

### Molecular cloning, expression, and purification of recombinant EhSWP1

The complete ORF of *EhSWP1* (687 bp) was amplified from cDNA obtained from the hepatopancreas of EHP-infected shrimp (GenBank accession no. MG015710). PCR conditions were the same as previously described in the RT-PCR analysis section. The gene was inserted between *Nco*I and *Xho*I restriction sites of the pET28 expression vector (Novagen, Queensland, Australia) to generate a pET28a_SWP1 that was transformed into *Escherichia coli* BL21 Star (DE3). Positive clones were analyzed by restriction endonuclease analysis and confirmed by DNA sequencing (Macrogen, South Korea). A selected positive clone was grown in Luria-Bertani (LB) medium and induced with 0.4 mM IPTG (isopropyl β-D-1-thiogalactopyranoside) at 37 °C for 4 h. Bacterial cells were harvested by centrifugation at 14,000× *g* at 4 °C for 10 min.

To purify recombinant EhSWP1, a bacterial cell pellet was re-suspended with 1× PBS and broken by sonication. After that, the mixture was centrifuged at 14,000× *g* at 4 °C for 15 min. The supernatant was collected and mixed with protein lysis buffer (50 mM NaH_2_PO_4_, 300 mM NaCl, 10 mM imidazole; pH 8) prior to loading onto a Ni^2+^-NTA affinity column (Qiagen, Hilden, Germany). Protein and Ni^2+^-beads were incubated for 1 h at 4 °C. Then, the column was washed with 10 column volumes of wash buffer (50 mM NaH_2_PO_4_, 300 mM NaCl, 20 mM imidazole; pH 8). The purified recombinant EhSWP1 was eluted with elution buffer (50 mM NaH_2_PO_4_, 300 mM NaCl, 250 mM imidazole; pH 8). All protein fractions were analyzed by 12.5% SDS-PAGE. Protein concentrations were measured using Bradford reagent (BioRad, California, USA). The purified recombinant EhSWP1 was dialyzed against 1× PBS at 4 °C overnight.

### Polyclonal antibody production and Western blot analysis

To produce a polyclonal antibody against EhSWP1, purified recombinant EhSWP1 was sent to a commercial antibody production facility (Singapore Advanced Biologics, Singapore) to immunize rabbits. After the third immunization, rabbit sera containing anti-EhSWP1 antibody were collected and specificity of anti-EhSWP1 antibody was tested by Western blot analysis.

For Western blot analysis, purified recombinant EhSWP1 was separated by 12.5% SDS-PAGE and transferred to a nitrocellulose membrane. The membrane was blocked with blocking solution (5% skim milk in 1× PBS) for 1 h at room temperature (RT) followed by incubation with 1:2000 anti-EhSWP1 antibody or naïve rabbit serum as a negative control in blocking solution for 1 h at RT. After six washes with PBST buffer (1× PBS, 0.05% Tween 20), 1:3000 goat anti-rabbit IgG conjugated with alkaline phosphatase enzyme (GAR-AP) was applied for 1 h at RT and later washed with PBST buffer three times. Finally, colorimetric signals were developed by BCIP/NBT phosphatase substrate (Millipore, Massachusetts, USA).

### Immunofluorescence analysis (IFA)

Purified EHP spores were added onto poly-lysine coated slides and dried at RT overnight. The spores were fixed with 4% paraformaldehyde at RT for 15 min followed by washing with 1× PBS three times and permeabilized with 1% Triton X-100 at RT for 30 min. Next, the spores were blocked with blocking reagent (10% normal goat serum, 5% bovine serum albumin in 1× PBS) at RT for 90 min prior to incubation with 1:100 anti-EhSWP1 antibody in blocking reagent at RT for 3 h. The negative control group was incubated with naïve rabbit serum. After six washes, 1:200 goat anti-rabbit antibody conjugated with Alexa 488 (GAR-Alexa488) was added and incubated at RT for 1 h. 1:2000 TO-PRO-3 dye was used to stain nuclei for 5 min at RT. Finally, slides were mounted with 50% glycerol. The fluorescence signals were examined using a confocal laser scanning microscope (Olympus FV10i-DOC).

### Immunoelectron analysis (IEM)

Purified EHP spores and EHP-infected hepatopancreatic tissue were fixed with 4% paraformaldehyde and 0.5% glutaraldehyde in 0.1 M sodium cacodylate buffer pH 7.2 for 1 h at RT and then rinsed with 1× PBS four times. The samples were dehydrated with a graded ethanol series including 50%, 75% and 100% for 15 min each step followed by permeabilizing and embedding in LR-white (Electron Microscopy Sciences, Pennsylvania, USA). LR-white was polymerized at 65 °C overnight. Next, ultrathin sections were placed onto 300-mesh nickel grids. For immunostaining, the grids were blocked with blocking solution (1% bovine serum albumin, 0.02% NaN_3_, 5% normal goat serum in 1× PBS) for 2 h at RT and incubated with 1:10 anti-EhSWP1 antibody in blocking solution for 2 h at RT. For the negative control group, naïve rabbit serum was used instead of anti-EhSWP1 antibody. After six washes with 1× PBS, 1:100 anti-rabbit IgG conjugated with 10 nm gold particles (Sigma-Aldrich, Massachusetts, USA) in blocking solution was applied onto the grids for 1 h at RT and then washed with distilled water. Finally, the grids were counterstained using 4% uranyl acetate for 2 min and gold particles were examined under a Hitachi H7100 transmission electron microscope (TEM) at an accelerating voltage of 100 kV.

### Site-directed mutagenesis of *EhSWP1*

Basic amino acid residues of all three HBMs found in *EhSWP1* gene were mutated into glycine or serine using a gene synthesis facility (Synbio Technologies, USA). *EhSWP1*(B→G) contained the following mutations: R11G, K12G, K14G, K15G, R35G, K36G, R38G, K62G, H63G, H65G and H66G, while *EhSWP1*(B→S) contained mutations R11S, K12S, K14S, K15S, R35S, K36S, R38S, K62S, H63S, H65S and H66S. After that, mutated *EhSWP1* genes were subcloned into the pET28a expression vector (Novagen, Queensland, Australia). Protein expression and purification were followed as previously described for EhSWP1 WT.

### Heparin bead binding and competition assays

Purified recombinant EhSWP1 (20 μg) or 20 μg of bovine serum albumin (Sigma-Aldrich, Massachusetts, USA) were mixed with 50 μl of pre-equilibrated heparin-sepharose beads (50% slurry) with 1× PBS (GE Healthcare, Buckinghamshire, UK) at 4 °C for 1 h with radial rotation. For the heparin competition assay, various concentrations (0.1, 1, 10 and 100 mg/ml) of porcine heparin sodium salt (Sigma-Aldrich, Massachusetts, USA) were mixed with recombinant EhSWP1 prior to incubation with heparin-sepharose beads. The beads were then washed three times with 1× PBS (5 min incubation in each washing step). Proteins were eluted with elution buffer (2 M NaCl in 1× PBS). All protein fractions were visualized by 12.5% SDS-PAGE with Coomassie blue staining. To quantify the level of heparin binding, the intensity of the protein band was quantified using Scion Image software (Version 4.0). Level of heparin binding in the group without competitor (0 mg/ml heparin group) was used for normalization.

### Statistical analysis

The percentages of heparin binding were expressed as means ± standard error of the mean (SEM). The difference between each heparin concentration was tested using one-way ANOVA.

## Results

### Identification and characterization of EhSWP1

To better understand the pathogenesis of EHP, a search for its potential virulence factors was carried out by analyzing the EHP genome [40] and categorizing genes according to their functions (Table 1). Putative EHP virulence factors included genes involved in host cell invasion, spore attachment, energy parasitism and host cell manipulation. To infect their host cells, microsporidia have been reported to utilize SWPs as a recognition system [17, 45]. Herein, we describe identification of a spore wall protein, EhSWP1 (EHP00_686). The full-length coding sequence of *EhSWP1* is 687 bp encoding a deduced protein of 228 amino acids (GenBank accession no. MG015710), with a molecular mass of 27 kDa and a theoretical isoelectric point of 8.45.

**Table 1:** Putative virulence factors of EHP

Function	Gene
Host cell invasion and spore attachment	Polar tube proteins (PTPs)
	Spore wall proteins (SWPs)
	Endochitinases
	Chitin synthases
Energy parasitism	ADP/ATP transporters
Host cell manipulation	Mitogen-activated protein kinases
	Transferases
	Splicing machineries

### Phylogenetic analysis of EhSWP1

**Fig. 1:**
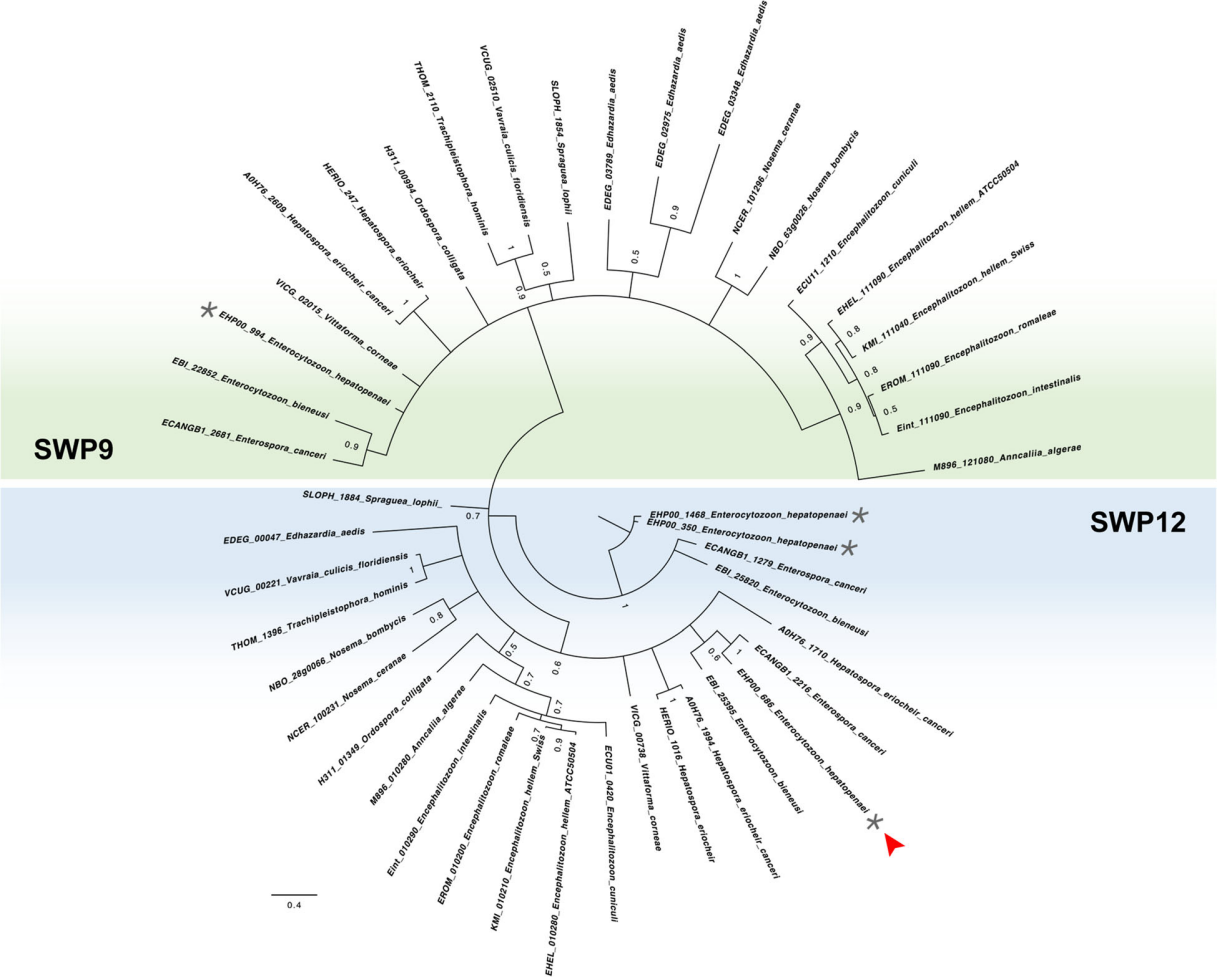
Sequence analysis of EHP SWPs. Bayesian Inference phylogenetic analyses of proteins that were grouped in the same ORTHOMCL ortholog clusters with *Enterocytozoon hepatopenaei* proteins annotated as SWP in its genomic assembly. The dotted line arcs delineate the two distinct clades made up of SWP12 and SWP7 proteins. *E. hepatopenaei* proteins are indicated with asterisk (*). Red arrowhead represents EhSWP1 (EHP00_686). Numbers on nodes are Bayesian posterior probability values

**Fig. 2:**
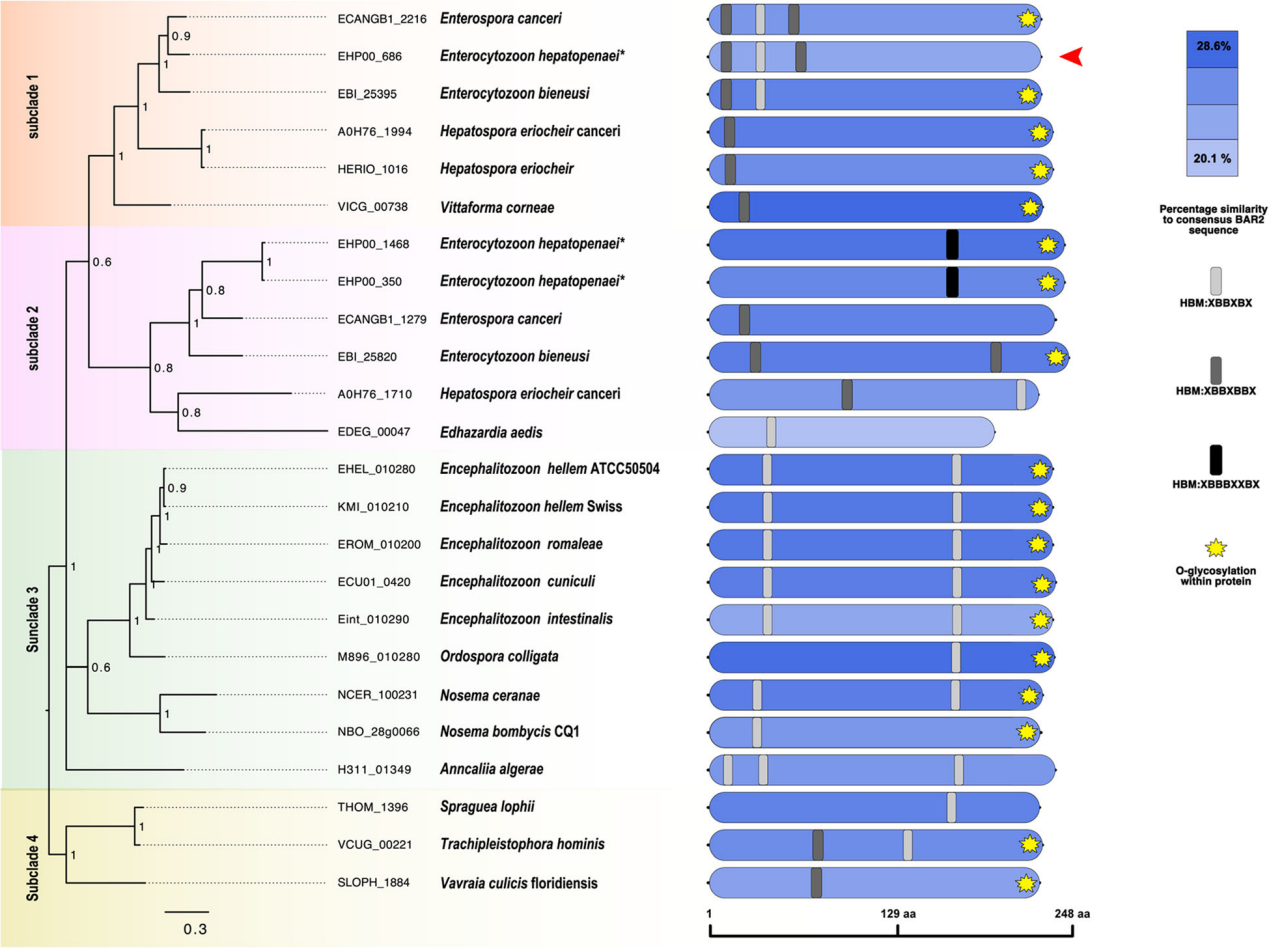
Domain organization of EHP SWPs. Bayesian inference analyses of proteins in the SWP12 clade. Blue rounded rectangles represent conservation of the BAR2 domain across this clade with their hues reflecting their level of similarity to the BAR2 HMM seed consensus sequence. Hues assigned with the heat map module in R STUDIO. Conservation of Heparin Binding Motifs (HBMs) is represented with small grey curved rectangles. Subclades have been delimitated with different background colors. Numbers on nodes are Bayesian posterior probability values. EHP SWPs are indicated with asterisk (*) and red arrowhead represents EhSWP1 (EHP00_686)

An initial NCBI word search for SWP in the genomic assembly of EHP identified proteins with the following accession numbers OQS53864.1 (EHP00_686), OQS55031.1 (EHP00_944), OQS55055.1 (EHP00_1468) and OQS53422.1 (EHP00_350). In this study, we focused on EHP00_686, which we named EhSWP1. Our orthology analyses revealed that EhSWP1 (EHP00_686) and EHP00_350 were in a different ortholog cluster from EHP00_944 (Fig. 1). Interestingly, EHP00_1468 did not cluster with any other microsporidian protein used in this analysis despite having a 98% identity to EHP00_350 in our BLASTP search results. Bayesian inference (BI) analyses resulted in a tree that had representative proteins from the two ortholog clusters in two distinct clades (Fig. 1). The clade in which EhSWP1, EHP00_350 and EHP00_1468 clustered contained other microsporidian proteins that were predominantly annotated as SWP12, whereas EHP00_944 was grouped within a clade containing proteins that were predominantly annotated as SWP7. Both SWP12 and SWP7 were previously described in *Nosema bombycis* [24, 46] and they were used as the name of the clades in this study. The phylogenetic relationship between these clades was however poorly supported statistically in both Bayesian and maximum likelihood (ML) analyses (Fig. 1). Apart from *Nematocida* species, all other microsporidian species used in this analysis were represented by at least a single protein in both the SWP12 and SWP7 clades (Fig. 2).

An initial search for functional domains in proteins belonging to the SWP12 clade showed that some of them encoded a Bin-amphiphysin-Rvs-2 (BAR2) domain. Unlike proteins in the SWP12 clade, a scan for functional domains for proteins in the SWP7 clade showed that they did not share a common functional domain. When aligned against the consensus sequence of BAR2 HMM seed sequences, proteins in the SWP12 clade showed amino acid similarity ranging between 20–29 %. The BAR2 domain of *Saccharomyces cerevisiae* protein YP148 that was one of the seed sequences used in the construction of the BAR2 HMM was 29% similar to the consensus sequence (data not shown). Proteins belonging to *V. corneae* and members of the family Encephalitozoonidae displayed the highest amino acid similarity (Fig. 2). Contrary to MOTIF SCAN results that predicted the BAR2 domains of most SWP12 clade proteins to be located in their C-terminus, amino acid pair-wise alignment analyses showed that the BAR2 domain spanned the entire length of these proteins.

A regular expression search predicted all proteins in the SWP12 clade to encode, at least, a single heparin binding motif (HBM) whereas only M896_121080 (*Ordospora colligata*), EDEG_03348 (*Edhazardia aedis*), NBO_63g0026 (*Nosema bombycis*) and ECANGB1_2681 (*Enterospora canceri*) in the SWP7 clade encoded heparin binding motifs. In this study, three HBMs were identified at the N-terminus of EhSWP1 (EHP00_686). The position of the first XBBXBBX HBM was conserved only in the family Enterocytozoonidae whereas that of the second XBBXBX HBM was conserved among most but not all microsporidian species (Fig. 2). Interestingly, the position of the third XBBXBX HBM was conserved only in EhSWP1 and ECANGB1_2216. EHP00_350 and EHP00_1468 were the only proteins in this analyses that contained the XBBBXXBX HBM signature sequence.

EhSWP1 was among the few proteins that were not predicted to possess any O-glycosylation sites (see yellow stars in Fig. 2). While all proteins in the SWP12 clade were predicted to contain phosphorylation sites, none of them were positive for signal peptide sequences, GPI anchoring and transmembrane domains.

### Gene expression pattern of *EhSWP1* during an infection

**Fig. 3:**
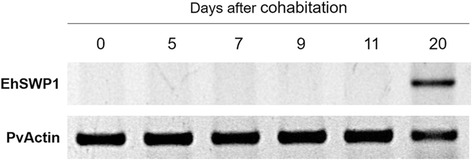
EhSWP1 transcripts can be detected 20 days after cohabitation. The mRNA expression of EhSWP1 was analyzed by RT-PCR using RNA template extracted from hepatopancreatic tissue of naïve shrimp cohabitated with EHP-infected shrimp. Shrimp samples were collected at 0, 5, 7, 9, 11 and 20 days after the start of cohabitation between naïve shrimp and EHP-infected shrimp. The actin gene of *P. vannamei* (PvActin) was used as an internal control

To investigate the expression pattern of the *EhSWP1* gene, single step RT-PCR analysis was performed using cDNA generated from hepatopancreatic tissue of naïve shrimp collected on days 0, 5, 7, 9, 11 and 20 after cohabitation with EHP-infected shrimp. Positive RT-PCR amplicons for the *EhSWP1* gene were detected in the naïve shrimp at 20 days after the start of cohabitation (Fig. 3). However, subsequent testing using a more sensitive nested RT-PCR method revealed a low level of *EhSWP1* at 11 days after cohabitation (Additional file 1: Figure S1). This indicated that a measurable level of infection was evident much earlier than 20 days and that progression of the infection was not very rapid.

### Immunolocalization of EhSWP1

**Fig. 4:**
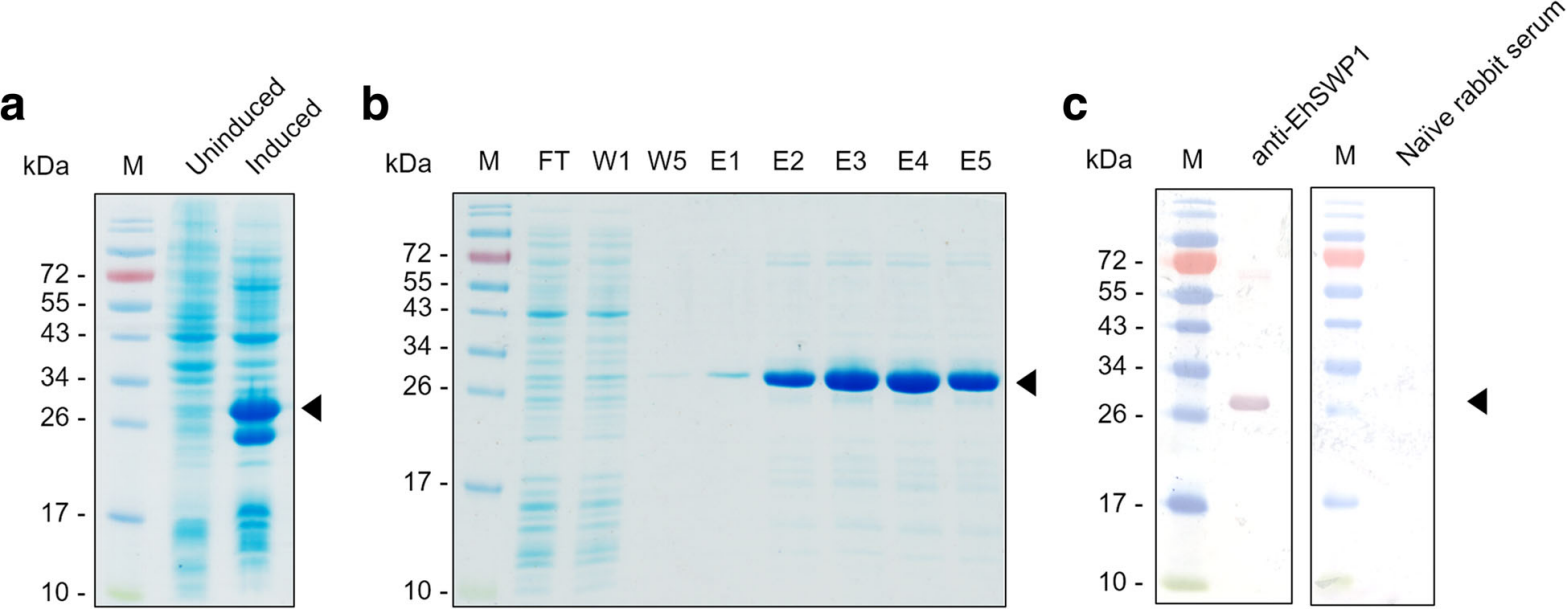
Expression, purification and Western blot analysis of recombinant EhSWP1. **a** SDS-PAGE gel compared between uninduced *E. coli* BL21 star(DE3) cells and induced *E. coli* cells with 0.4 mM IPTG. **b** SDS-PAGE gel showing purified recombinant EhSWP1 obtained using Ni^2+^-NTA affinity chromatography. Lane FT shows the flow-through fraction; W1 and W5 are wash fractions 1 and 5, respectively; E1-E5 are elution fractions 1–5. **c** Immunoblot of recombinant EhSWP1 probed with rabbit anti-SWP1 antibody and naïve rabbit serum as a negative control. The recombinant EhSWP1 band is indicated by a black arrow. Lane M: protein molecular weight marker

Purified EhSWP1-His_6_ was expressed in *E. coli.* After induction with IPTG, a 27 kDa overexpressed band of recombinant EhSWP1 was observed (Fig. 4a). Purification with Ni^2+^-NTA affinity chromatography showed that purified protein was found in fractions 2 to 5 (Fig. 4a: lanes E2-E5) after elution with 300 mM imidazole (Fig. 4b). Later, purified protein was pooled prior to immunization of rabbits to generate polyclonal antibody against EhSWP1. Specificity of the antibody was tested by western blot analysis (Fig. 4c). The result revealed a strong positive band at 27 kDa that was consistent with the size of recombinant EhSWP1 (Fig. 4c). Thus, anti-EhSWP1 antibody specifically bound to recombinant EhSWP1 and was suitable for localization studies.

**Fig. 5:**
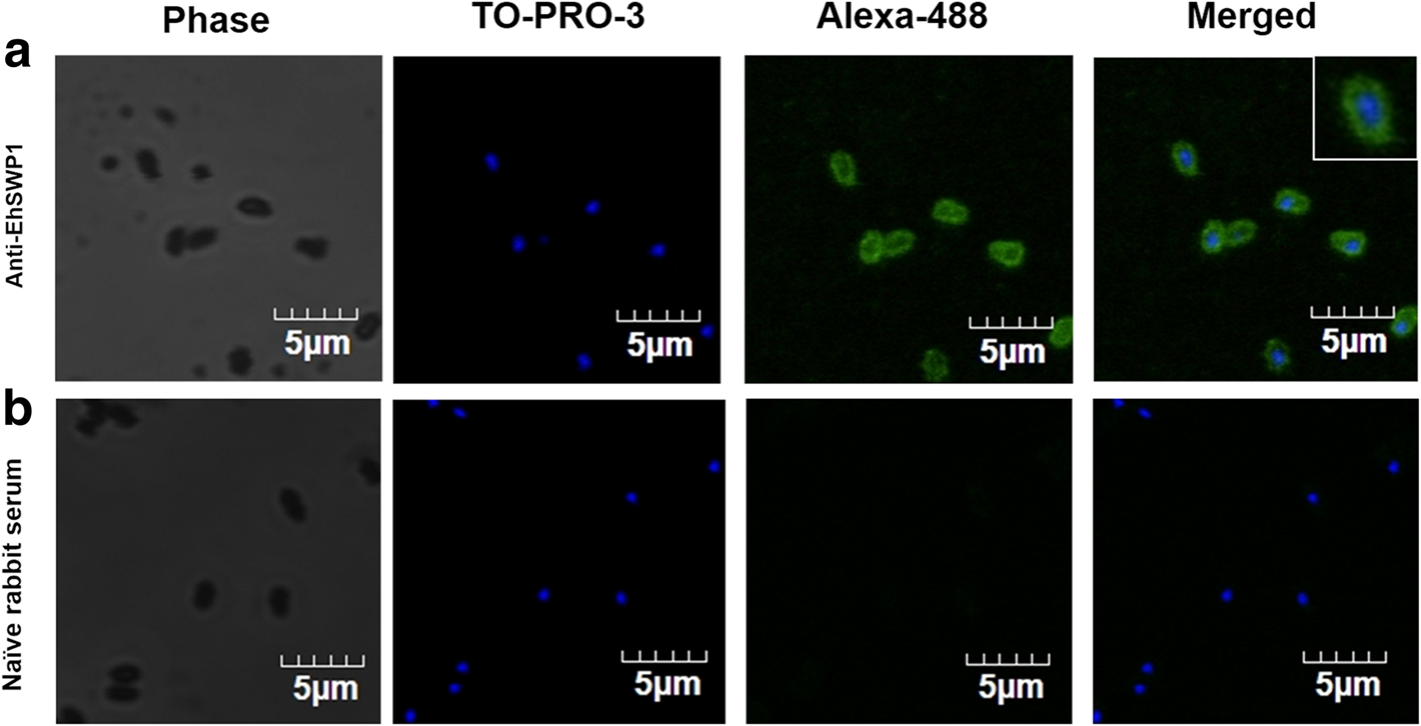
Immunofluorescence analysis (IFA) reveals the localization of EhSWP1 in the spore wall. Green fluorescence (Alexa-488) indicates the localization of EhSWP1 in mature spores. Phase shows the phase contrast micrographs. TO-PRO-3 was used to stain the nuclei of EHP spores (blue fluorescence). **a** Anti-SWP1 antibody was used as a primary antibody. A higher magnification is shown in the inset. **b** Naïve rabbit serum was used a negative control

When rabbit anti-EhSWP1 was used to perform immunofluorescence analysis (IFA) with purified spores of EHP, green fluorescence from Alexa-488 dye revealed that EhSWP1 was localized on their periphery (Fig. 5a). TO-PRO-3 dye (blue fluorescence) revealed the nucleus within EHP spores (Fig. 5). For the negative control group, no green fluorescence was detected (Fig. 5b). Therefore, these data confirmed that EhSWP1 was an EHP spore-wall protein.

**Fig. 6:**
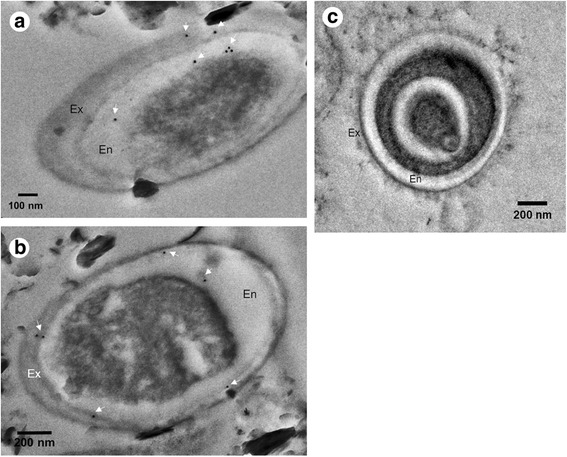
Subcellular localization of EhSWP1 using Immunoelectron analysis (IEM). **a**, **b** Electron micrographs reveal the localization of EhSWP1. Exposure to anti-SWP1 antibody followed by GAR-IgG conjugated with 10 nm gold particles revealed immunogold particles (indicated with white arrows) indicating the presence of EhSWP1 in the exospore and endospore of EHP. **c** Negative control probed with naïve rabbit serum shows no immunogold signals. *Abbreviations*: Ex, exospore layer; En, endospore layer

Further immunoelectron analysis (IEM) to determine the subcellular localization of EhSWP1 revealed immunogold particles in both the exospore (Ex) and endospore layers (En), but not in the plasmalemma (Fig. 6a, b) or in the spore cytoplasm. No immunogold particles were found in the negative control group (Fig. 6c).

### Interaction of EhSWP1 with heparin and a competition assay

**Fig. 7:**
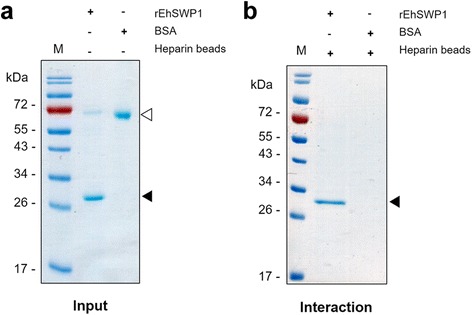
Recombinant EhSWP1 binds to heparin in vitro. **a** SDS-PAGE gel showing input recombinant EhSWP1 (black arrow) and bovine serum albumin (BSA, white arrow) prior to mixing with heparin sepharose beads. **b** SDS-PAGE gel showing fractions eluted with 2 M NaCl and indicating that only EhSWP1 (black arrow) was captured and eluted from the heparin beads

Since sequence analysis revealed that EhSWP1 had three heparin binding motifs at its N-terminus, preliminary assays were carried out to test its ability to bind with heparin in vitro. When recombinant EhSWP1 and BSA (Fig. 7a) were incubated with heparin beads, only recombinant EhSWP1 (but not BSA) was bound and subsequently eluted (Fig. 7b). It was possible but unlikely that the band in Fig. 7b arose from a contaminant *E. coli* protein of the same electrophoretic mobility as recombinant EhSWP1, but this possibility was eliminated in the following experiment below.

**Fig. 8:**
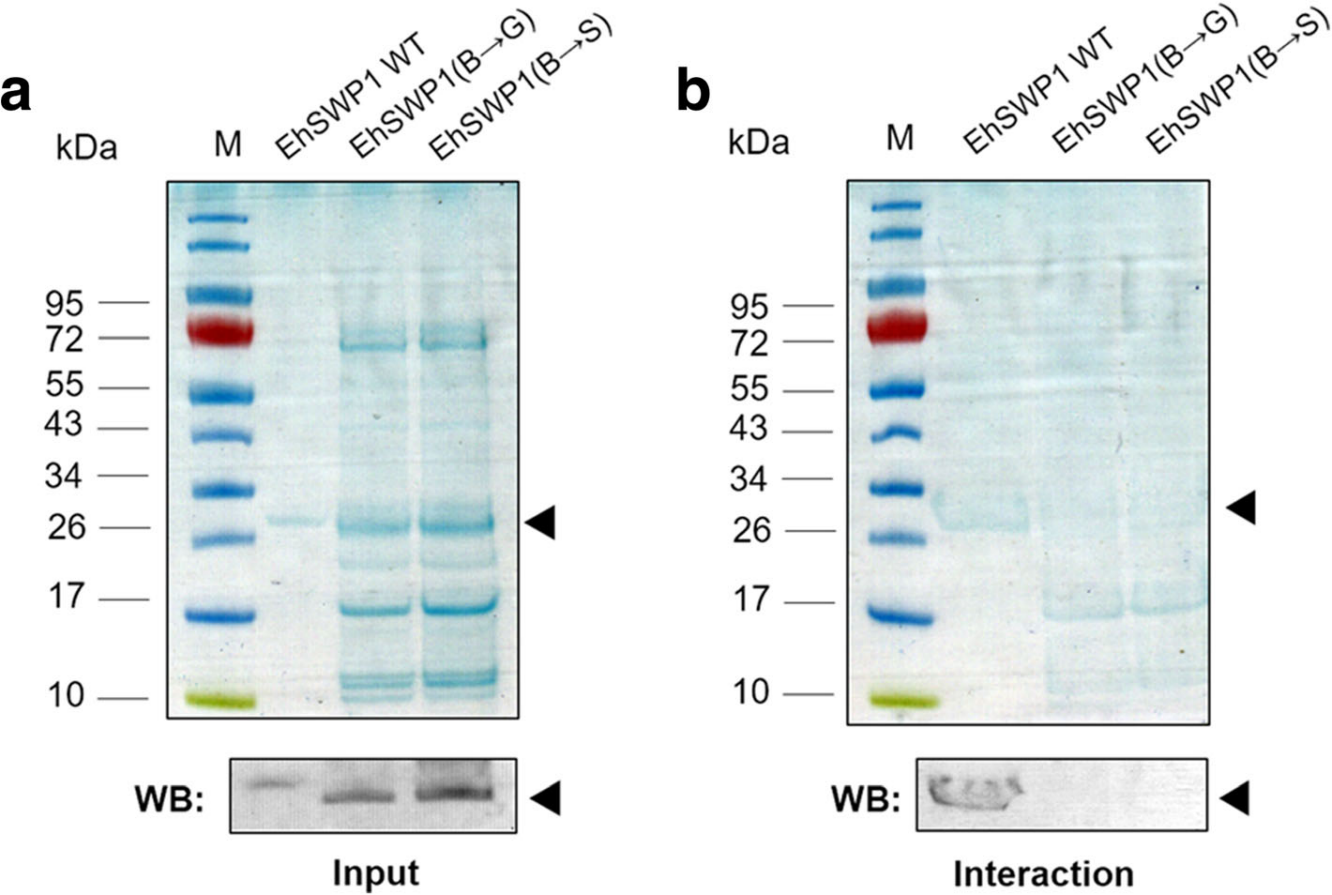
EhSWP1-HBM mutants fail to bind to heparin beads. **a** SDS-PAGE gel showing input proteins EhSWP1 WT, EhSWP1(B→G) and EhSWP1(B→S) (black arrow) with molecular weights of 27 kDa, 26 kDa and 26 kDa, respectively. **b** SDS-PAGE gel showing elution fractions after incubation with heparin sepharose beads and revealing that only EhSWP1 WT (black arrow) was captured and eluted from the beads. Lower panels (indicated as WB) are western blots probed with anti-EhSWP1 antibody to confirm protein identity as EhSWP1 (black arrows)

In addition, since previous studies [47, 48] showed that basic residues in HBM are important for its binding activity to negatively-charged heparin, we used in vitro mutation to determine whether the function of HBM in EhSWP1 was related to heparin binding. Positively charged amino acids arginine, lysine and histidine in the three HBMs were mutated to uncharged glycine [EhSWP1(B→G)], or to partially negative serine [EhSWP1(B→S)]. Due to the substitution of larger side chains with smaller side chains, EhSWP1(B→G) and EhSWP1(B→S) were1-kDa lower in molecular weight than EhSWP1 wild type (EhSWP1 WT). Mutation to alanine was also carried out. However, almost all of the overexpressed alanine mutant proteins were insoluble (data not shown). Input proteins for the binding experiment are shown in Fig. 8a. After incubation of EhSWP1 WT, EhSWP1(B→G) and EhSWP1(B→S) with heparin beads followed by elution with 2 M NaCl, only EhSWP1 WT was found in the elution fraction, not EhSWP1(B→G) or EhSWP1(B→S) (Fig. 8b). Western blot results using the anti-EhSWP1 antibody confirmed that only EhSWP1 WT did bind to heparin, while EhSWP1(B→G) and EhSWP1(B→S) did not (Fig. 8). This result confirmed that EhSWP1-HBMs are important for heparin binding. Since all three recombinant proteins were produced using the same *E. coli* expression system, the negative western blot results for EhSWP1(B→G) or EhSWP1(B→S) (Fig. 8b) also eliminated the unlikely possibility that the band in Fig. 7b and the immunopositive band in Fig. 8b arose from a contaminant *E. coli* protein of the same electrophoretic mobility as recombinant EhSWP1.

**Fig. 9:**
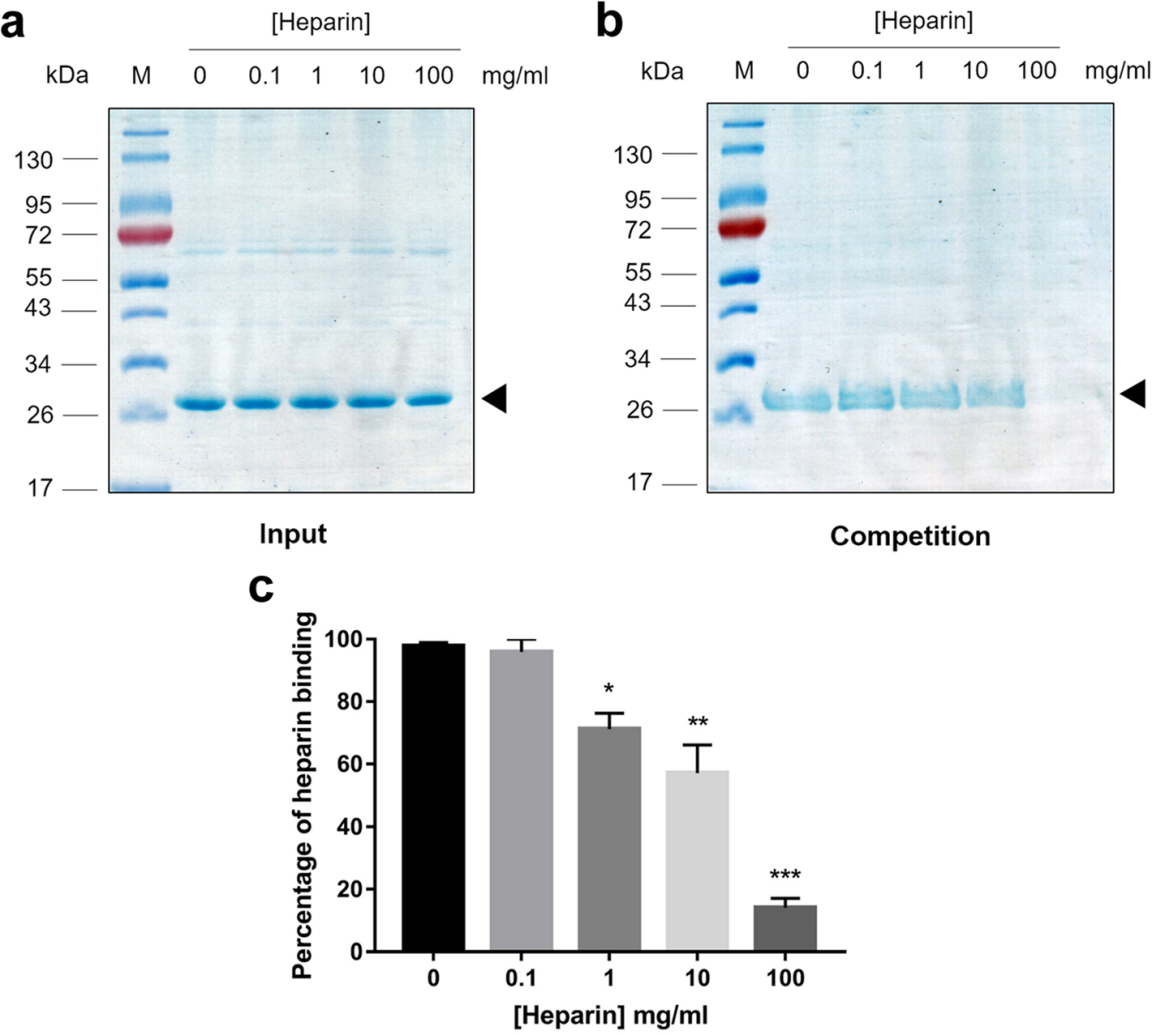
Heparin competition assay. **a** SDS-PAGE gel showing input recombinant EhSWP1 (black arrow) with different concentrations of soluble porcine heparin. **b** SDS-PAGE gel shows elution fractions after 1 h competition and revealing that binding of EhSWP1 (black arrow) to heparin beads was blocked at 100 mg/ml. **c** Bar graph showing percentage of heparin binding quantified from the protein bands from 3 with replicates gels (Additional file 2: Figure S2). Error bars indicate the mean ± SEM. Level of heparin binding at 0 mg/ml was used for normalization. *P ≤ 0.01; **P ≤ 0.001; ***P ≤ 0.0001

To confirm specificity of the binding, competition assays using soluble heparin were carried out. By pre-incubating four different concentrations of soluble heparin with recombinant EhSWP1 prior to mixing with heparin-sepharose beads, it was shown that 10 mg/ml of soluble heparin could reduce the binding by more than 40% (Fig. 9, Additional file 2: Figure S2). Increasing the soluble heparin to 100 mg/ml reduced the binding by 84% (Fig. 9c). However, there was no reduction in binding when there was no exogenous heparin or heparin at 0.1 mg/ml (Fig. 9c). This result suggests that exogenous heparin can inhibit the interaction of EhSWP1 with heparin in a dose dependent manner and that heparin is indeed an EhSWP1 binding partner.

## Discussion

### Diversity and phylogeny of spore wall proteins

The microsporidian infection process is unique compared to that of other intracellular parasites [49, 50]. Their spores possess a special organelle called a polar tube that is extruded to pierce host cell membranes and serve as a conduit to transfer the infectious spore contents (sporoplasm) into the host cell cytoplasm [16]. However, microsporidia require relatively close proximity to host cells for the first step of infection [17, 45]. Previous studies have revealed that SWPs are important in the attachment of microsporidian spores to their hosts [17, 18].

Orthology clustering and phylogenetic analyses performed in this study identified the four proteins annotated as SWPs in EHPs genomic assembly [40] to fall under two distinct clades of microsporidian SWPs, SWP12 and SWP7. Signature sequences of HBMs are well characterized, namely XBBXBX, XBBXBBX, XBBBXXBX and XBBBXXBBBXXBBX, where X represents a hydrophobic amino acid and B represents a positively charged amino acid [48, 51, 52]. Although XBBXBX and XBBXBBX HBMs appeared to be highly conserved across the SWP12 clade in our analysis, their exact positioning and enrichment was specific to microsporidian families and sometimes, species (Fig. 2). In light of the importance of SWP HBMs in parasite-host tethering [18, 24], this family/species-specific HBM positioning and enrichment perhaps reflect the different host environs and cell types with which these proteins have evolved to interact. Our phylogenetic analysis suggests there was a duplication of the *SWP12* gene in the common ancestor of species belonging to the family Enterocytozoonidae, with positional conservation of HBMs only being maintained in subclade 1 (Fig. 2). This duplication event, unique to the Enterocytozoonidae, hints at the importance of this particular protein in the life-cycle of species within this family. Gene duplication is known to facilitate innovation in genomes by allowing the duplicate gene to develop new functional properties via the accruement of non-deleterious mutations, a process referred to as neofunctionalization. Finally, our analyses corroborated previous research that predicted NbSWP12 (NBO_28g0066) and *E. intestinalis* EnP1 to contain 1 and 2 HBMs, respectively [18, 24].

Our alignment results suggest that the BAR2 domain is conserved across all proteins that clustered within the SWP12 clade. Known functions of this domain include membrane shaping and signalling control processes, but its role in microsporidian proteins is yet to be elucidated [53]. The conservation of this domain in the SWP12 clade, however, alludes to its importance in the function of SWP12 proteins [24].

### Expression profiles of spore wall proteins

Expression profiles of SWPs vary in different microsporidian species. Feeding of fourth instar silkworm larvae with mulberry leaves contaminated with *N. bombycis* spores showed that NbSWP5, NbSWP12 and NbSWP15 were expressed on day 3 post-infection [22, 24, 25]. In contrast, transcripts of NbSWP11 appeared at a low level on day 1 post-infection and gradually rose until day 7 [23]. Moreover, starvation treatment of third instar nymph locusts followed by feeding with *A. locustae* spores revealed that *Aloc*SWP2 expression was detected on day 9 after spore inoculation [30]. Our cohabitation study between naïve shrimp and EHP-infected shrimp showed that EhSWP1 transcripts were observed only at 20 days after the start of cohabitation. However, by using RT-PCR followed by nested-PCR analysis specific to the *EhSWP1* gene, a low level of expression was found at 11 days after cohabitation. The result may suggest that EHP requires at least 11 days to develop into mature spores. However, this needs to be confirmed by other analyses.

### EhSWP1: its role in host-cell tethering

**Fig. 10:**
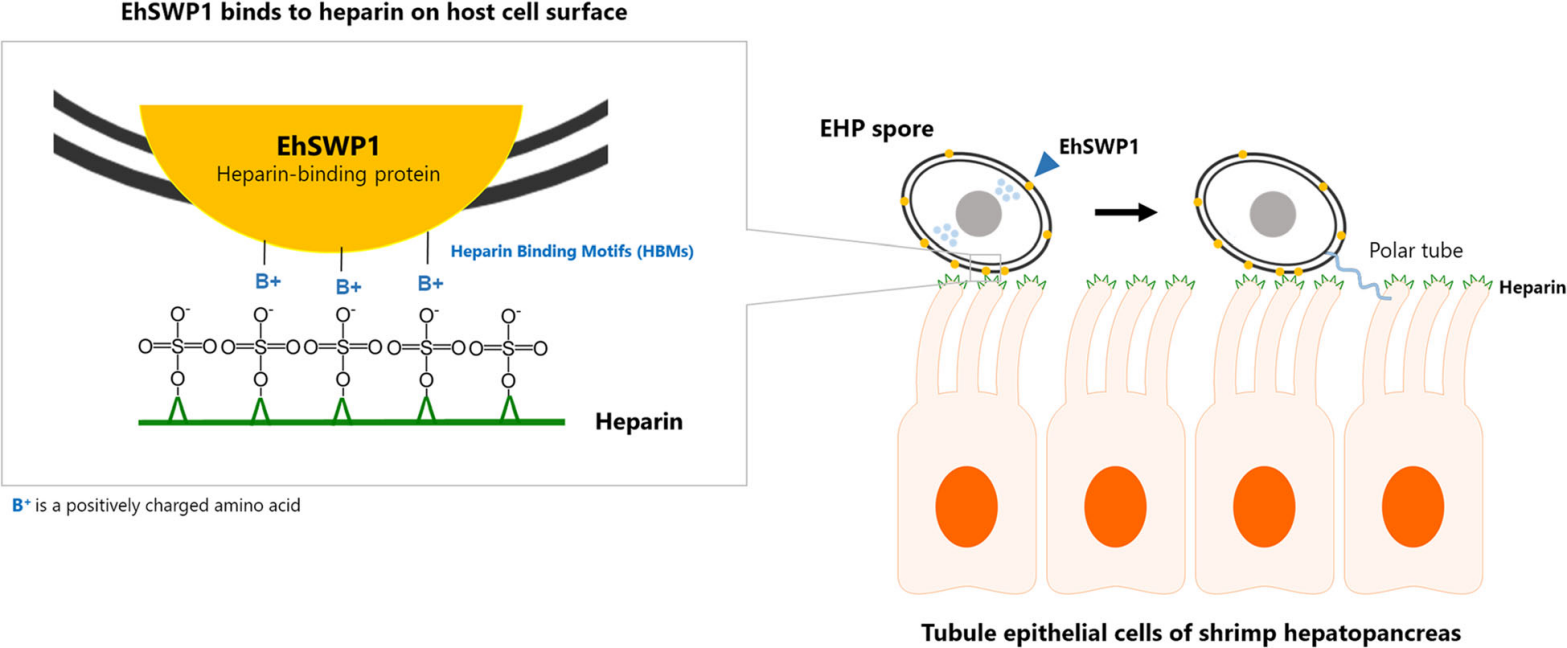
A schematic model of how EhSWP1 functions in host cell attachment. In order to invade shrimp cells, EHP must be in close proximity to tubule epithelial cells of shrimp hepatopancreas. From our results, we hypothesize that spores of EHP are attracted to the epithelial cells through the electrostatic interactions between positively charged residues (Arg, Lys and His) in the three HBMs of EhSWP1 and negatively charged heparin on cell surface. Once anchored, the EHP spores extrude their polar tube to pierce the host cell membrane and release sporoplasm into host cytoplasm where the next developmental stages occur

Heparin is a member of the GAG family and has been extensively studied in vertebrate species. A major function of heparin is to serve as a blood anticoagulant [54]. It is also used as an antithrombotic agent against heart and vascular thrombosis [55]. In mammals, heparin is mainly distributed in the lungs, intestine and liver [56]. Heparin is not only found in vertebrates, but also in invertebrates including crustaceans, molluscs, annelids, echinoderms and cnidarians [57]. However, there are very few studies on localization of heparin in organs and cell types. In the northern quahog clam, heparin was found at the proximal to epithelial surfaces of cells in the intestine, palp and siphon [58]. For shrimp, there has been no study on heparin distribution. However, heparin has been successfully extracted from the cephalothorax (where the gills, heart, intestine and hepatopancreas are located [59]) in the red-spotted shrimp *P. brasilliensis* and the Pacific white shrimp *P. vannamei* [60, 61]. Transcriptomic analysis of the hepatopancreas of *P. vannamei* showed that genes involved in the GAG biosynthesis pathway were active [62] and suggested that heparin might be present in the hepatopancreas. In this study, we showed that EhSWP1 could bind to heparin using the in vitro heparin binding assay. From immunofluorescence and immunoelectron analyses of EHP spores, we also showed that EhSWP1 is localized in both the exospore and endospore layers, similar to what has been previously described for SWPs from other microsporidians including EiEnP1, NbSWP9 and NbSWP26 [18, 27, 63]. The results support our hypothesis that EHP uses EhSWP1 to bind to heparin of target cells in shrimp hepatopancreatic tissue (Fig. 10) [33, 34].

It is not only EHP that utilizes heparin for attachment to host cells. Other intracellular parasites such as *Trypanosoma cruzi* also use heparin-binding proteins for host cell recognition. Incubation of its epimastigote stage with heparin and heparin sulfate can inhibit parasite binding to immobilized heparin and also inhibit parasite binding to midgut epithelial cells of their insect vectors [64]. In the malarial parasite *Plasmodium falciparum*, BAEBL protein binding to erythrocytes was disrupted by addition of soluble heparin [65]. The competition assay presented here showed that soluble heparin inhibited interaction between EhSWP1 and immobilized heparin beads in a dose dependent manner and suggests that heparin would inhibit EhSWP1 binding to shrimp host cells via their surface heparin.

Since there is no EHP infection model in hepatopancreatic cell cultures or any immortal shrimp cell line, in vivo tests of spore adherence could not be carried out but should constitute a future goal to confirm whether exogenous soluble heparin could reduce or inhibit EHP spore attachment to host cells. Similar tests would also show whether or not the antibody against EhSWP1 could reduce spore adherence. From previous studies, anti-EcEnP1 antibody inhibited spore adherence by 56% [18], while anti-NbSWP16 antibody reduced adherence by 20% [25]. Such in vivo assays with host cells are required to fully understand the function of EhSWP1.

## Conclusions

In summary, this is the first report on characterization of a spore wall protein from the microsporidian *E. hepatopenaei* (EhSWP1). It is present in both the exospore and endospore layers of mature spore walls and it has been shown to bind with heparin, indicating a possible role in attachment to host cells via surface heparin as an early step in the host cell infection process and constituting an important role in virulence (Fig. 10). This knowledge may lead to the development of novel therapeutics to combat to EHP infection.

## Additional files

Additional file 1: **Figure S1.** Transcriptional pattern of EhSWP1 using one-step RT-PCR and nested RT-PCR analysis of RNA template from naïve shrimp cohabitated with EHP-infected shrimp. (TIFF 497 kb)

Additional file 2: **Figure S2.** Experimental replicates of the heparin competition assay. (a) replicate II and (b) replicate III. (TIFF 984 kb)

## Abbreviations

EHP: *Enterocytozoon hepatopenaei*
EhSWP1: Spore wall protein 1 of EHP
HBM: Heparin binding motif
ORF: Open reading frame
ppt: parts per trillion
