# Supplementary figures and images for: Identification, characterization and heparin binding capacity of a spore-wall, virulence protein from the shrimp microsporidian, Enterocytozoon hepatopenaei (EHP)
Source: Parasit Vectors. 2018 Mar 12;11:177. doi: 10.1186/s13071-018-2758-z (PMC5848443; doi:10.1186/s13071-018-2758-z)

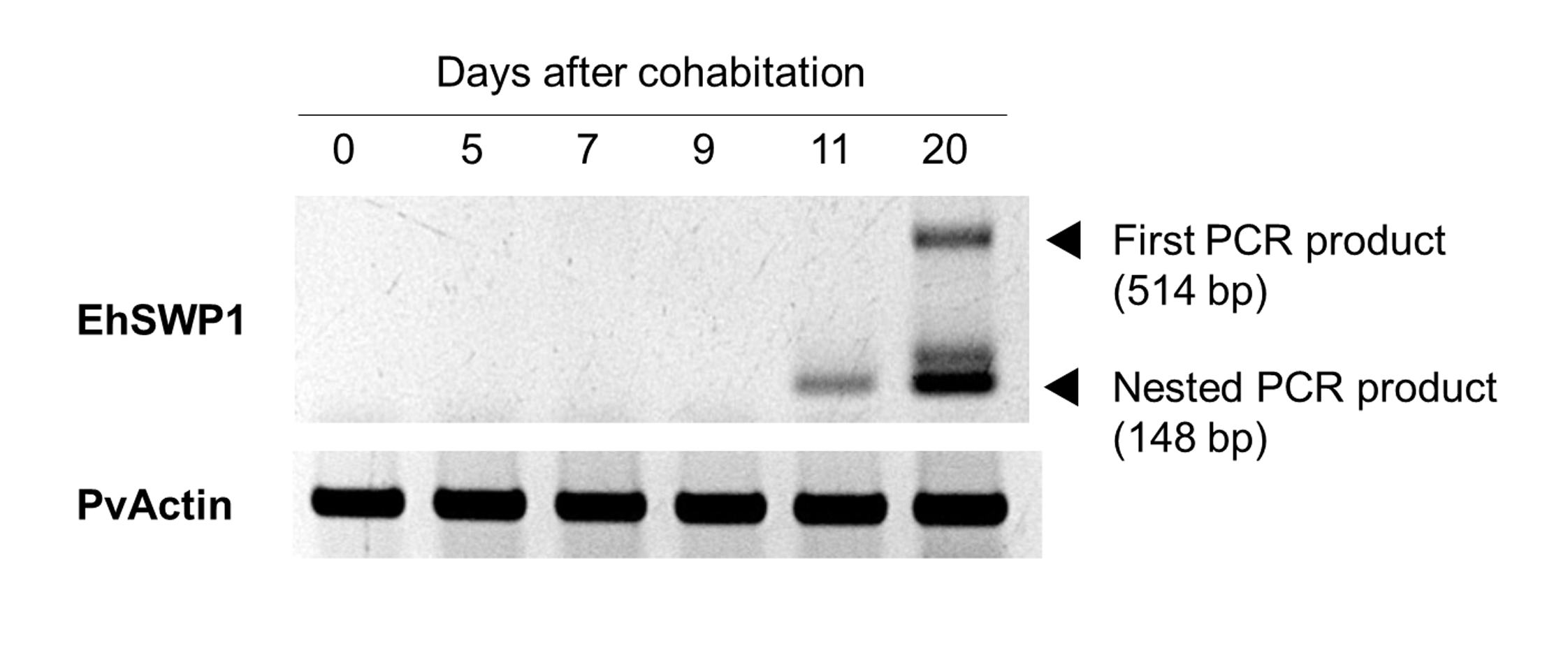

Supplement: Supplementary file 1 — Figure S1. Transcriptional pattern of EhSWP1 using one-step RT-PCR and nested RT-PCR analysis of RNA template from naïve shrimp cohabitated with EHP-infected shrimp. (TIFF 497 kb) [file 13071_2018_2758_MOESM1_ESM.tif]

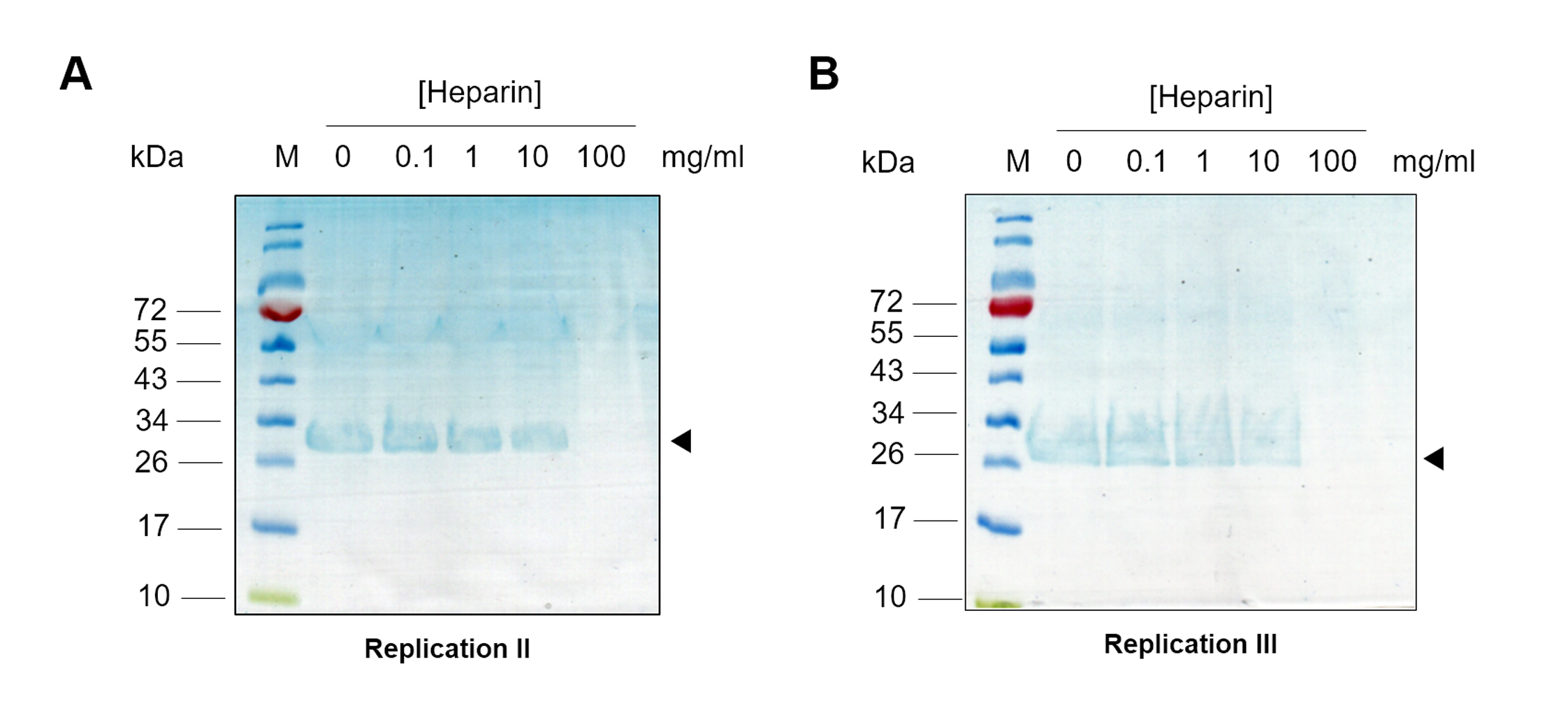

Supplement: Supplementary file 2 — Figure S2. Experimental replicates of the heparin competition assay. (a) replicate II and (b) replicate III. (TIFF 984 kb) [file 13071_2018_2758_MOESM2_ESM.tif]
